# *Mytilus galloprovincialis* as a smart micro-pump

**DOI:** 10.1242/bio.021048

**Published:** 2016-09-09

**Authors:** Fazil E. Uslu, Kerem Pekkan

**Affiliations:** Mechanical Engineering Department, Koc University, Istanbul 34450, Turkey

**Keywords:** Bivalves, Particle image velocimetry, Jet flow, Suction feeding, Micro pumps, Mussel filtration

## Abstract

Hydrodynamic performance of the marine mussel, *Mytilus galloprovincialis*, is studied with time-resolved particle image velocimetry. We evaluated inhalant flow, exhalant jet flow, suction performance and flow control capabilities of the mussels quantitatively. Inhalant flow structures of mussels are measured at the coronal plane for the first time in literature. Nutrient fluid is convected into the mussel by three-dimensional sink flow. Inhalant velocity reaches its highest magnitude inside the mussel mantle while it is accelerating outward from the mussels. We calculated pressure gradient at the coronal plane. As inhalant flow approaches the mussel shell tip, suction force generated by the inhalant flow increases and becomes significant at the shell tip. Likewise, exhalant jet flow regimes were studied for 17 mussels. Mussels can control their exhalant jet flow structure from a single potential core region to double potential core region or vice versa. Peak exhalant jet velocity generated by the mussels changes between 2.77 cm s^−1^ and 11.1 cm s^−1^ as a function of mussel cavity volume. Measurements of hydrodynamic dissipation at the sagittal plane revealed no interaction between the inhalant and exhalant jet flow, indicating energy-efficient synchronized pumping mechanism. This efficient pumping mechanism is associated with the flow-turning angle between inhalant and exhalant jet flows, ∼90° (s.d. 12°).

## Abstract (precis)

**Summary:** The common Mediterranean mussel*, Mytilus galloprovincialis,* efficiently generates low Reynolds number inhalant suction flow and unique time-dependent exhalant jet structures.

## INTRODUCTION

Suspension-feeding bivalves filter large volumes of water very efficiently through a variety of biological pumping and feeding characteristics (Jørgensen, 1955, 1982, 1966; Wright et al., 1982; Meyhöfer, 1985; Riisgård and Larsen, 1995, 2000, 2001; Riisgård et al., 2015). Among many alternative pumping configurations employed by the bivalves, mussels are classified as ‘hydrodynamic pumps’ due to their organized ciliary network and synchronized microscopic beating patterns (Bach et al., 2015). For example, the low Reynolds (Re) number particle retention mechanism of *Mytilus edulis* (blue mussel) is shown to be critical for internal flow performance (Jørgensen, 1983; Nielsen et al., 1993).

Ciliary structures of mussel gills have an important biological function, which is to generate flow circulation inside the mussel cavity. The internal flow is generated by the lateral cilia located at interfilament canals (Seo et al., 2014). Propulsion characteristics of cilia have been studied to understand how flow through the interfilament canals and frontal surface currents are generated at the micro-scale. Latero-frontal cilia located at the entrance of the interfilament canals can retain particles that are above 4 μm diameter (Riisgård and Larsen, 2001). Separated particles are sent to surface currents generated by frontal cilia (Nielsen et al., 1993). Frontal surface currents take the particles separated by latero-frontal cilia to the nutrient groove (Riisgård et al., 1996). While several research groups investigated the morphology and evolution of mussel gills in order to understand the flow mechanics through the interfilament canals and frontal surface currents (Brennen and Winet, 1977; Jørgensen et al., 1984; Gueron and Liron, 1992; Cannuel et al., 2009; Aksit and Mutaf, 2014), these studies lacked real-time flow measurements. This present study is unique in that it visualized and quantified the velocity field at the coronal plane during suction feeding through a particle image velocimetry (PIV) technique. As such, artificial cilia models were employed to understand the contribution of individual cilia to the flow circulation (Jonas et al., 2011; Chen et al., 2014). As highlighted in the present study, the ciliary propulsion mechanism of mussel gills is also crucial for the generation of external inhalant suction and the exhalant jet flow regimes.

In addition to internal flow performance, the external flow structures generated proximal to the bivalve mantle is hypothesized to be equally critical for the long-term sustained flow efficiency, non-interacting and simultaneously generated inhalant and exhalant jet flows without excessive hydrodynamic energy dissipation. The mantle shape, exhalant and inhalant siphons are the major functional components influencing the external flow performance. In earlier ‘qualitative’ investigations, using dyes as flow tracers, streamlines generated by the siphon components were visualized (Monismith et al., 1990; O'Riordan et al., 1995). In a quantitative study, Frank et al. (2008) employed PIV to compare the external velocity fields generated by five different bivalve suspension feeders without studying inhalant flow and its interaction with the exhalant jet. Likewise, Troost et al. (2009) only investigated inhalant flow fields created by three suspension feeders and presented detailed inhalant velocity field information through PIV. For the exhalant flow side, Riisgård et al. (2011) presented flow measurements focusing on the exhalant flow of *Mytilus edulis,* but without including detailed analyses on inhalant flow as performed in the present study. We hypothesized that the interaction between inhalant flow and exhalant jet is important; we thus conducted the present experimental campaign to investigate the degree of external flow interaction between inhalant and exhalant flow streams. To evaluate if any flow interaction exists, we studied exhalant jet flow and inhalant flow simultaneously through multiple measurement planes

In summary, previous investigations that focused on suspension feeders did not study the jet flow angle between the inhalant and exhalant flow, which is hypothesized to be important for efficient feeding. To our knowledge, this is also the first literature to suggest that the mantle shape may be important for the optimal inflow and exit flow; to investigate this we acquired flow fields at multiple planes. Furthermore, we studied how mussels can dynamically change their exhalant jet flow type over time to demonstrate the tremendous changes in shape and maximum velocity values of the exhalant jet flow.

## RESULTS

### External flow structures

**Fig. 1.**
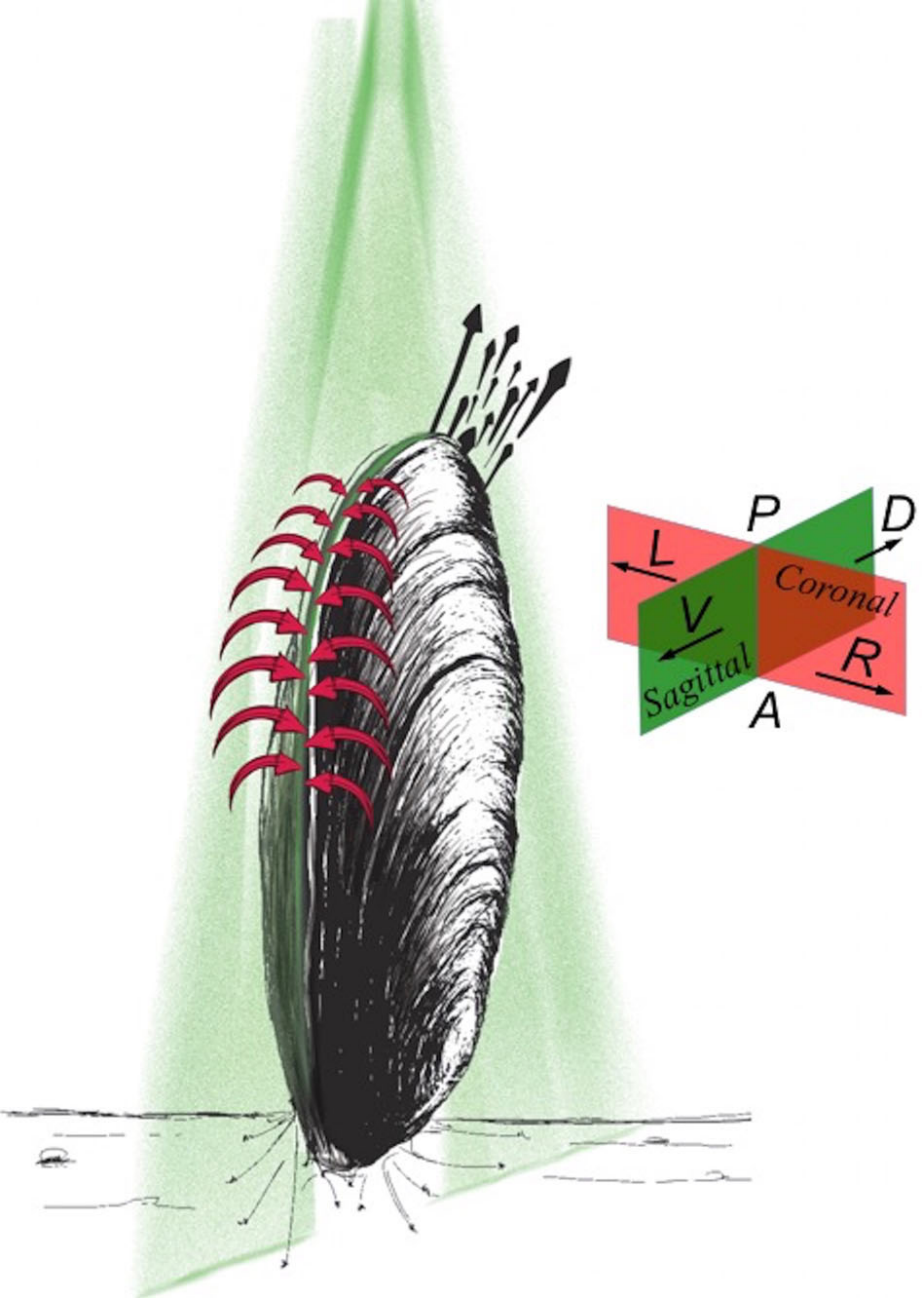
**A cartoon representation of the main flow structures generated by *M. galloprovincialis* as observed during our experiments.** Sink-type inhalant flow is shown with red streamlines. Black arrows illustrate the exhalant jet flow. A laser sheet oriented along the sagittal plane is plotted in green. Body directions and anatomical planes where the measurements are performed are represented in the right side of the mussel. The sagittal plane is represented with a green plane showing ventral (V) and dorsal (D) directions. The coronal plane is represented with a red plane showing left (L) and right (R) directions. The posterior (P) and anterior (A) sides are also labeled. This plot is generated through qualitative dye-visualization experiments in addition to the PIV.

Mussels can simultaneously generate the exhalant jet and inhalant suction. The associated main flow structures are illustrated qualitatively in Fig. 1, based on our flow visualization experiments. Both the exhalant jet regime and inhalant flows are distinct for most flow states. The exhalant flow resembles a low Re number jet flow, which emerges as a single or double potential core region. Determination of an exact Re number is challenging due to the dynamic changes in the exhalant siphon effective orifice area, but are estimated to range between 50 and 500. Whereas for the inlet, a sink-type inhalant flow is observed with lower Re numbers (Re<90). Sink flow occurs along line absorbing fluid inwards and the inhalant siphon corresponds to the line where the fluid is absorbed. The radial flow area spanned by the exhalant siphon is significantly smaller than the inhalant siphon area, as also reported by Troost et al. (2009), resulting in higher exhalant jet velocities.

### Inhalant flow field

**Fig. 2.**
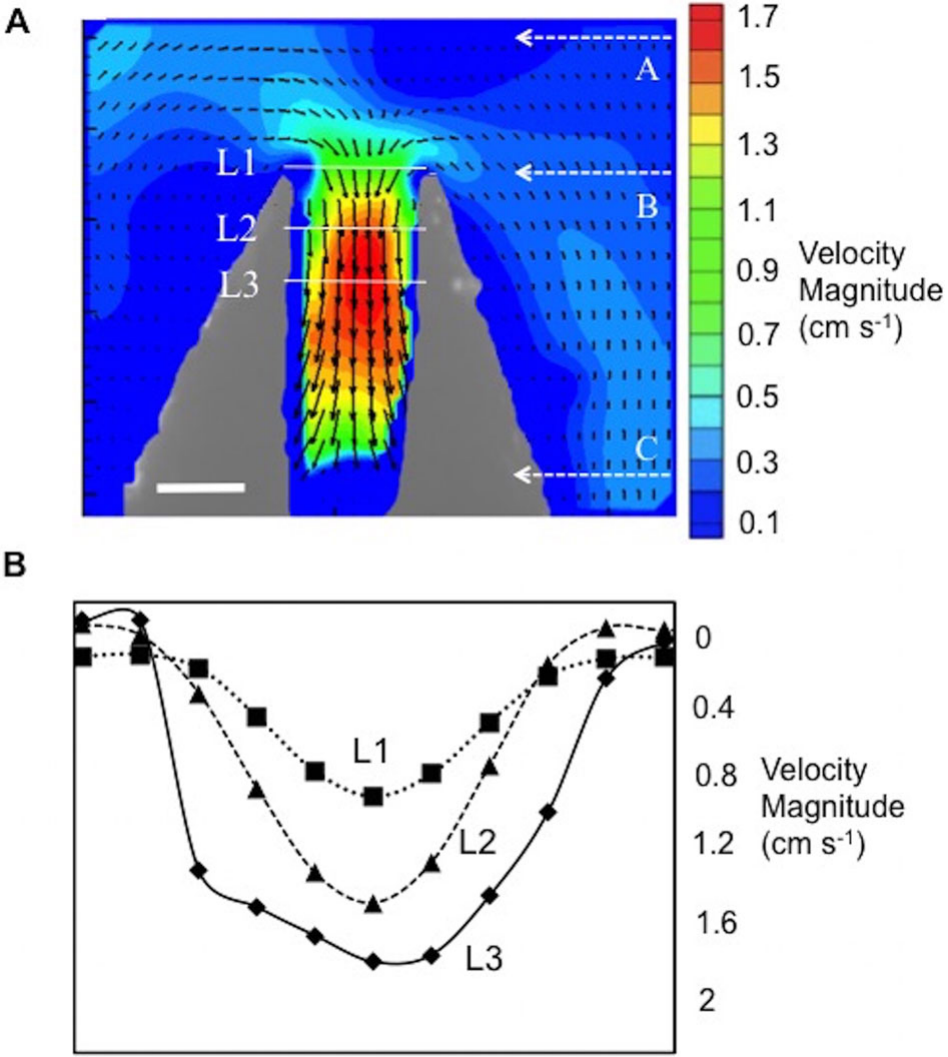
**Vector field measurements at the coronal plane during suction feeding are presented.** (A) PIV velocity field of mussel (volume of 34 cm^3^) inhalant flow along the coronal plane. Dashed lines represented with A, B and C labels show the locations referred to in Fig. 3. Scale bar is 0.5 cm. (B) Velocity profiles at L1 (squares with dotted line), L2 (triangles with dashed line) and L3 (diamonds) are plotted. Horizontal axis spans the entire length of lines L1, L2 and L3. Velocities sampled at L1, L2 and L3 correspond to the velocity magnitude of posterior-anterior velocity components. Corresponding lines; L1, L2 and L3 are shown in A.

The velocity field acquired in the coronal plane is presented in Fig. 2. Suction flow starts to accelerate proximal to the mantle curvature. While the exact acceleration region outside the mussel changes from subject to subject, primary acceleration is induced inside of the mussel, and the flow reaches a maximum velocity of 1.8 cm s^−1^ (Fig. 2).

**Fig. 3.**
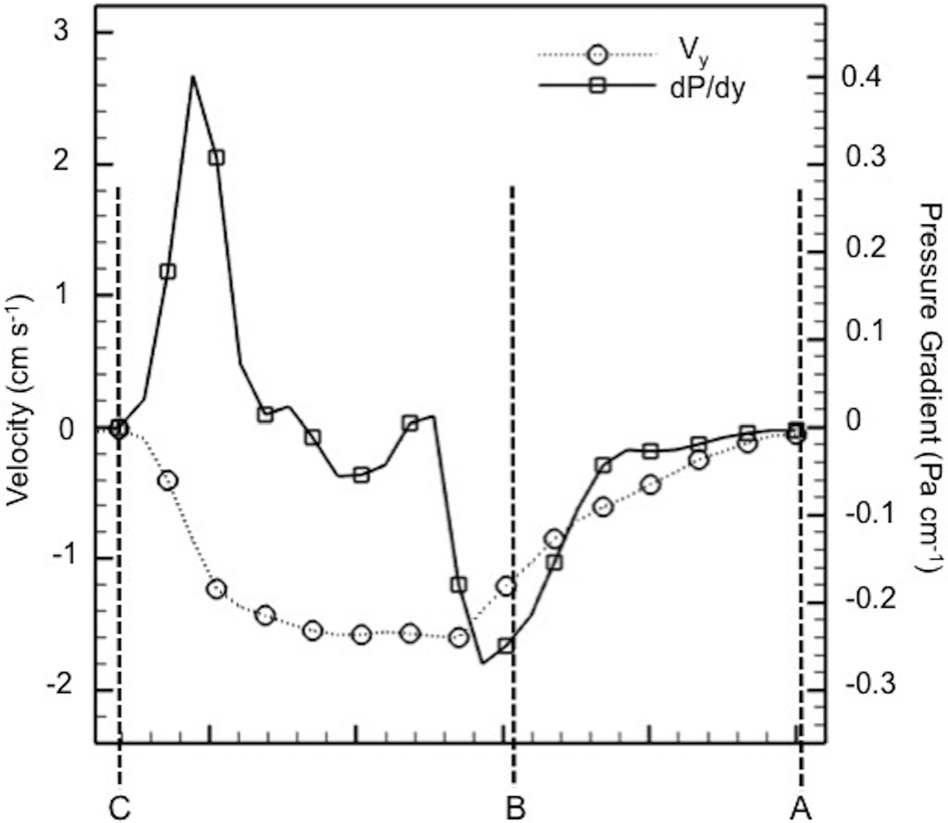
**Average pressure gradient (dP/dy) and average inflow velocity distribution (V_y_) is plotted along the inhalant suction streamline.** *y*-direction corresponds to posterior-anterior direction. Horizontal axis is shown with A, B, and C letters. Locations of A, B and C are denoted in Fig. 2A. Location of B corresponds to the point where inhalant flow comes inside the mussel cavity.

Velocity profiles (magnitudes) that are acquired at three axial locations are plotted in Fig. 2B, illustrating the inflow velocity development. Velocity increases with decreasing distance to the mussel and reaches the global maximum inside the mussel. All velocity profiles are roughly parabolic, but the peak velocity increases close to the mantle core and reaches its maximum value of 1.8 cm s^−1^. Nutrient water coming inside the mussel shell through suction feeding diffuses to both the left and right sides of the mussel's interior. Fig. 3 displays the relationship between the velocity and pressure gradient at the centerline of the coronal plane. When velocity reaches its spatial maximum, the corresponding pressure gradient becomes zero inside the mussel mantle; in turn, the pressure gradient that corresponds to the suction force reaches its maximum at the entrance of the mussel cavity.

### Time-dependent exhalant jet flow regime

**Fig. 4.**
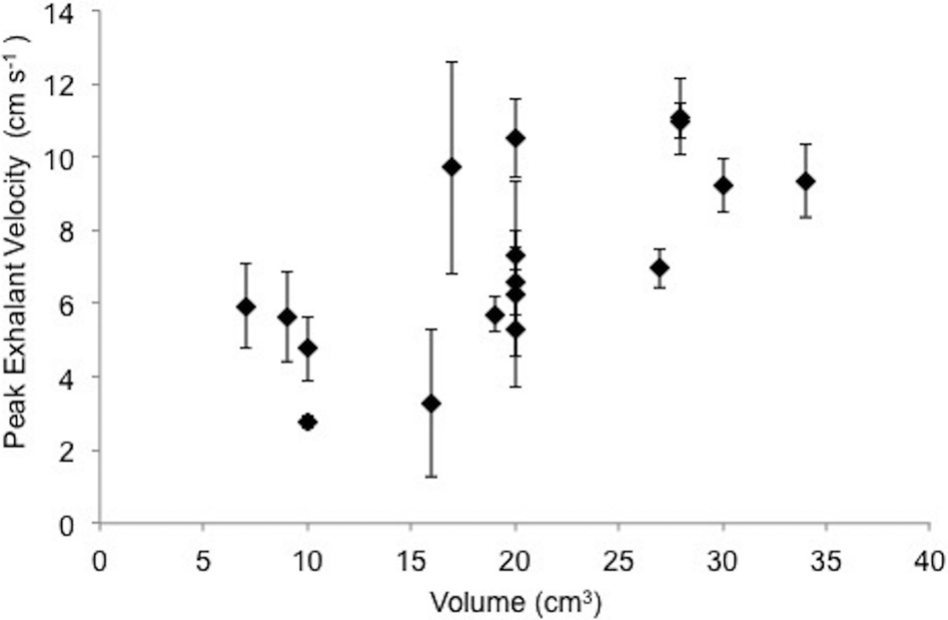
**Peak exhalant velocities of *M. galloprovincialis* are plotted as a function of mantle cavity volume.** A linear fit formula is obtained as *y*=0.2244*x*+2.7161 having an R^2^ value of 0.46. Error bars indicate one standard deviation of peak exhalant velocities for each mussel during the time course of measurements (∼4 s).

The exhalent flow occurred in non-periodic long-duration cycles. The peak velocities of the exhalant flow cycle are plotted in Fig. 4 for different mussel cavity sizes. Each point corresponds to the maximum exhalant jet velocity value averaged over 100 image pairs recorded during a period of relatively steady flow. We observe that exhalant jet flow peak velocity increases with increasing volume (R^2^=0.46). The highest recorded value of the peak exhalant velocity is 11.1 cm s^−1^, corresponding to a mussel volume of 28 cm^3^. The smallest value of peak exhalant velocity is 2.77 cm s^−1^ with a volume of 10 cm^3^. As such, the samples are grouped in three based on their peak exhalant jet velocities (Table 1). These groups generate 4.78 cm s^−1^, 6.83 cm s^−1^ and 9.52 cm s^−1^ average peak velocity with volume between 7-10 cm^3^, 16-20 cm^3^, and 27-34 cm^3^, respectively.

**Table 1.**
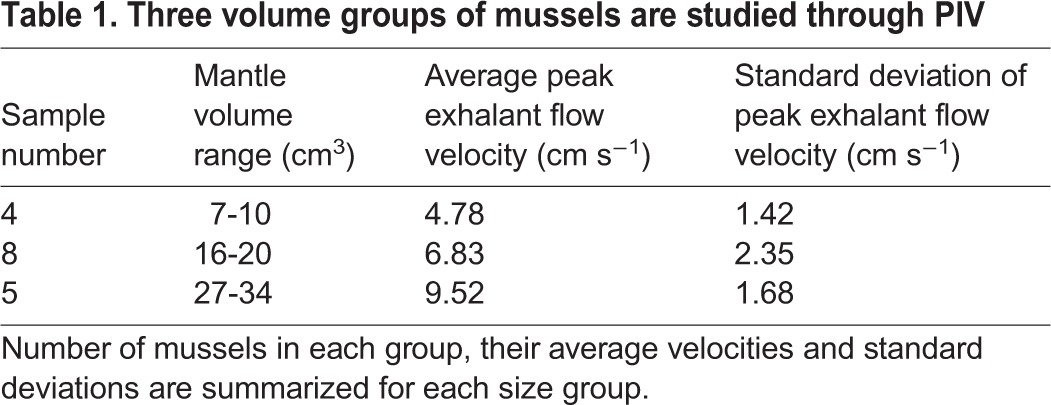
**Three volume groups of mussels are studied through PIV**

**Fig. 5.**
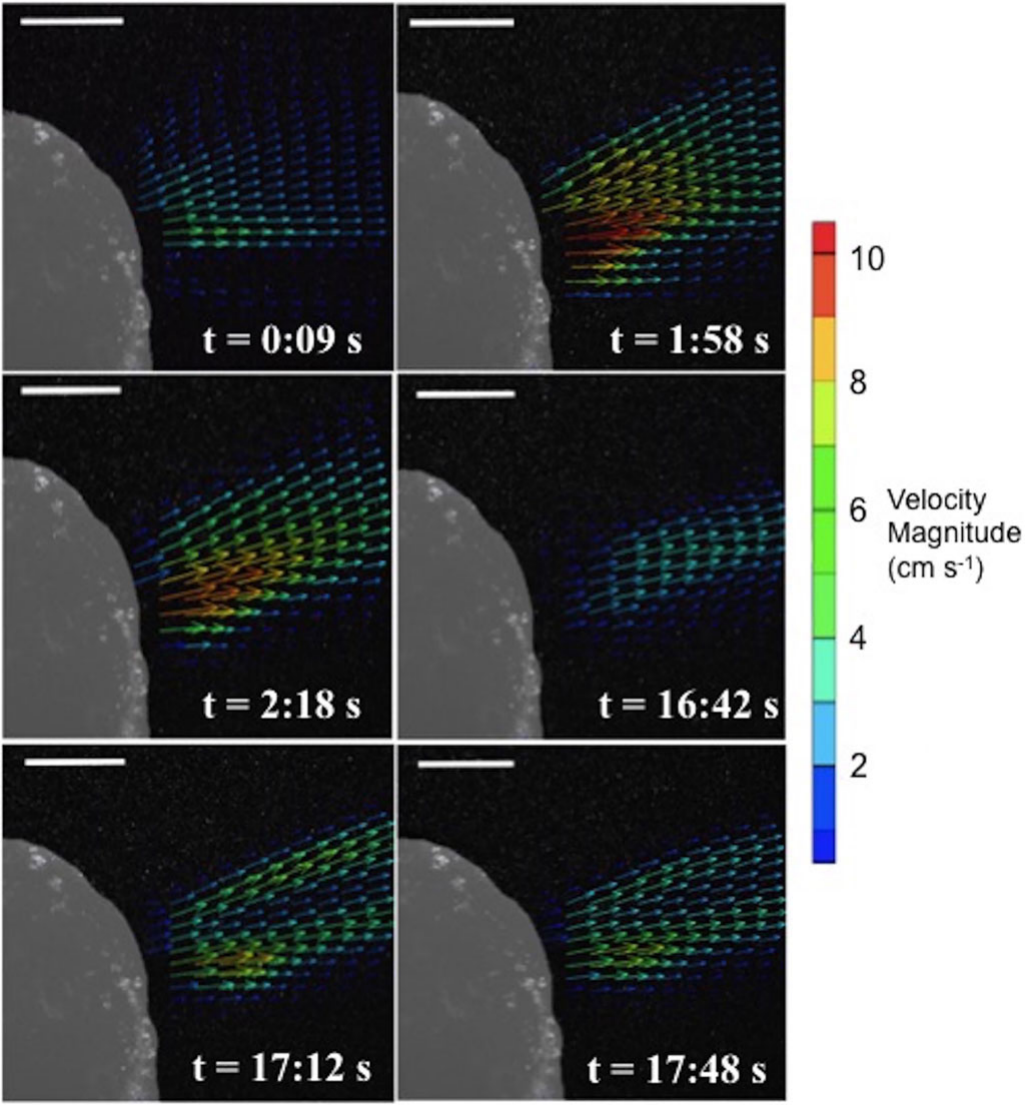
**Jet flow types observed during a typical exhalant flow cycle of the mussel.** These exhalant jet flow configurations are observed at different time points. The mussel (volume of 28 cm^3^) generates a single potential jet core region, typically during the start of the exhalant cycle between 9 s and 16 min 42 s. Later, this jet is converted to a jet having a double potential core region, likely modulated through the flexible siphon apparatus at time point of 17 min 12 s. Scale bars represent 1 cm.

Fig. 5 presents the consecutively recorded instantaneous exhalant jet flow fields for a mussel having a volume of 28 cm^3^. While a continuous stream of data is available, selected velocity fields are plotted to illustrate the observed exhalant jet flow structures. There was no periodicity in the flow patterns. The exhalant jet flow stream typically has a single core region during the initial phase of the exhalant flow cycle. The mussel converts the exhalant flow from a single core region of jet to a double core region, as can be observed from the instantaneous velocity fields of the last two time points (Fig. 5). 53% of the mussels generated double core jet regions for a finite duration during the velocimetry measurements. The separation of the jets randomly changes with the shape of the exhalant siphon, as is the case for the mussel depicted in Fig. 5 across all time points. The peak velocity of the exhalant flow is 6.21 cm s^−1^ at the initial measurement time point. Peak velocity increases to 11.1 cm s^−1^. After a 14 min time lapse, the peak velocity dramatically decreases to 4.5 cm s^−1^. The largest decrease in peak velocity is observed at this time point because exhalant jet velocity is also decelerating at this instant. The mussel jet changes from the single-core to a double-core structure. Peak exhalant velocities at the time points shown in Fig. 5 are 6.21 cm s^−1^, 11.1 cm s^−1^, 9.95 cm s^−1^, 4.5 cm s^−1^, 8.6 cm s^−1^ and 7.5 cm s^−1^, respectively.

**Fig. 6.**
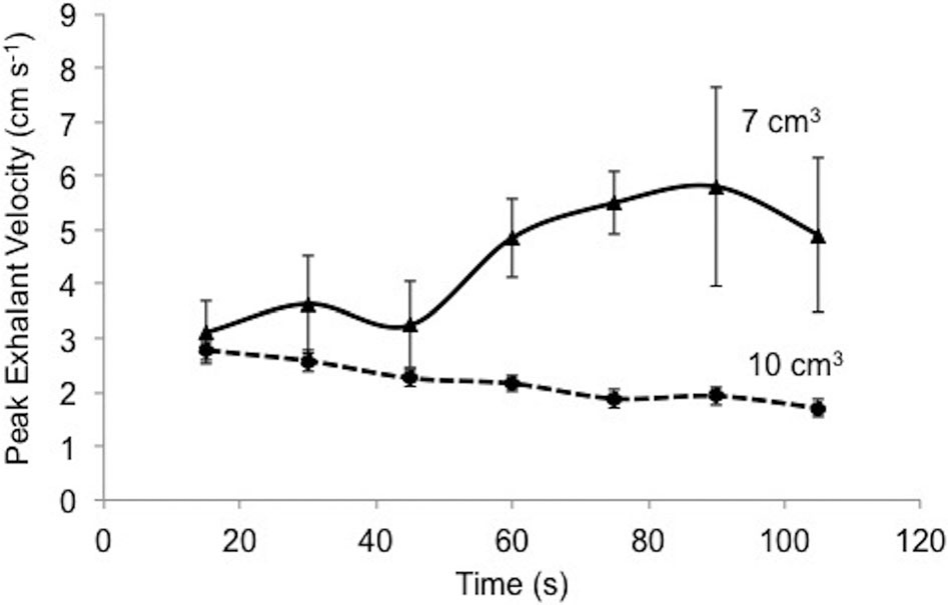
**The time-resolved peak exhalant flow cycles and peak velocity magnitudes are compared for two mussel samples having different sizes.** Peak exhalant flow velocities of two different mussels (volumes of 10 and 7 cm^3^) are tracked at the same time intervals. Error bars indicate one standard deviation of peak exhalant velocities for each mussel during the time course of measurements (∼4 s).

Fig. 6 displays the peak velocity recordings for exhalant flow as a function of time for two different sized mussels with volumes of 7 and 10 cm^3^ respectively. We obtained the peak velocity data at uniform time intervals. The peak exhalant flow velocity for the 7 cm^3^ mussel was initially recorded as 3.1 cm s^−1^ and increased by a factor of 2, to 5.8 cm s^−1^. The peak exhalant flow velocity for the 10 cm^3^ mussel was initially recorded as 2.77 cm s^−1^ and decreased to 1.7 cm s^−1^. We did not find a periodic signal produced by the peak velocity of exhalant flow. Each mussel may demonstrate differences in the exhalant jet flow behavior and the peak velocity of exhalant flow.

### Flow turning angle

**Fig. 7.**
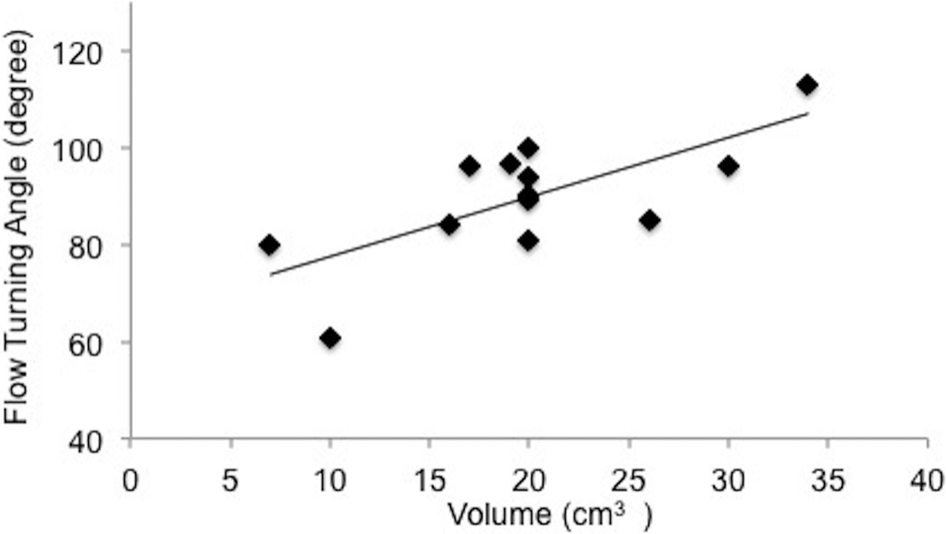
**Flow-turning angle (FTA) vs mussel volume is plotted.** FTA is defined between the exhalant jet and the direction of peak inhalant flow. Least-squares linear fit formula of flow turning angle is *y*=1.2296*x*+65.183 and R^2^ value is 0.51.

We define the flow turning angle (FTA) as the angle between the exhalant jet core direction and the inhalant maximum velocity vector. FTA is measured in the sagittal plane. For angle measurements, we selected the data set that has maximum peak velocity of exhalant jet flow for each mussel. Average FTA remains fairly constant at 90° with a standard deviation of 12° (Fig. 7).

### Energy dissipation rate

**Fig. 8.**
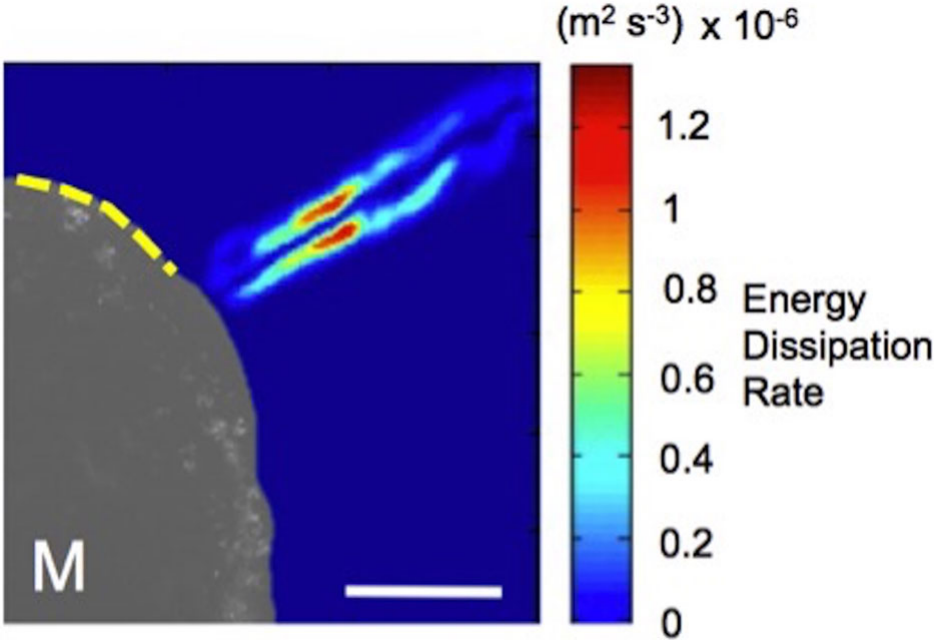
**Distribution of the energy dissipation rate of exhalant jet and inhalant flows plotted on the sagittal plane external to the mussel.** High dissipation rates are localized along the exhalant jet boundary layer. Yellow dashed line marks the region of inhalant suction flow, which is simultaneously generated with the exhalant jet. Scale bar is 1 cm. Mussel volume is 16 cm^3^. M, mussel.

We calculated the hydrodynamic dissipation to show that there is no interaction between the inhalant and exhalant jet flows for the measured FTA. The contour map of the time-averaged energy dissipation rate on the sagittal plane resembles a single core exhalant jet, as plotted in Fig. 8. The simultaneous inhalant flow region is also indicated with a yellow dashed line in Fig. 8, and is found to span a significantly larger flow area than the outflow. In spite of its close proximity, the simultaneous inhalant flow did not interfere significantly with the outflow jet, while the peak dissipation close to the inhalant flow region is slightly lower than the undisturbed jet boundary layer (see Fig. 8). Lower inflow speed due to a larger flow area and the FTA result in an optimal energy dissipation field. It is also observed that the dissipation rate of the developed exhalant jet is lower and becomes unstable. We observe fluctuations in instantaneous energy dissipation rate values of the exhalant flow, particularly along the jet boundary layer. Hydrodynamic dissipation map presents energy efficient pumping even tough the inhalant and exhalant jet flows are simultaneously generated and compete with each other.

## DISCUSSION

### Inhalant suction flow

Inhalant suction flow has a three-dimensional flow structure. In our experiments we minimized the out-of-plane flow velocity component (dorsal-ventral direction) by coinciding the coronal plane with the inhalant flow region which is closest to the posterior-dorsal direction side of mussel. This is a typical approach to present complex 3D flow structures and sufficient to understand the present flow regime.

The inhalant flow regime determines the nutrient seed capture performance. Inhalant flow also sets the characteristic operating point for the internal pumping apparatus of the mussel. The suction apparatus operates intermittently and provides a bolus of seawater to mussel gills for particle retention. In a recent study the internal flow circulation of *M. galloprovincialis* (Seo et al., 2014) was analyzed using phase-contrast magnetic resonance imaging (PC-MRI). PC-MRI sequences involve inherent averaging and could not capture the transient flow behavior as reported in the present study. As such, the detailed quantitative analysis of external flow structures provided in the present study supplemented the ‘internal’ PC-MRI measurements of mussels. The exhalant jet and inhalant suction velocity values measured through PIV are of the same order as reported by the PC-MRI measurements (Seo et al., 2014), which is critical for the validation of both experimental approaches. Furthermore, investigation of the inhalant flow along both the coronal and sagittal planes is novel, and provided crucial insight on the nutrient capture biomechanics. Nutrient capture biomechanics reveals important properties of suction feeding that is characterized by the suction force which is estimated from the velocity field measurements and pressure gradient calculations. The coronal plane provided a window of opportunity, where the velocity field deep inside the mussel's suction apparatus is measured through a quantitative measurement technique for the first time in the literature.

Suction feeding flow dynamics have been studied extensively in active feeding of vertebrates (Pekkan et al., 2016). The acceleration trend of the inhalant flow showed a similar behavior as the active suction feeding response of fish larvae operating at lower Re numbers (Yaniv et al., 2014). Inhalant flow accelerates while approaching the mussel, but gains its highest velocity value inside the mussel. After reaching its highest velocities, water is distributed to both the right and left gills (Fig. 2A), towards the interflamentary cavities powered by lateral cilia (Seo et al., 2014). Pressure gradient generated by the mussel determines the suction force acting on the food particles. The pressure gradient increases proximal and internal to the mussel mantle. Compared to the inefficient low Re number in larval fish feeding (Yaniv et al., 2014), the suction force in mussels influences a larger area proximal to the mussel mantle (Fig. 3) and is significantly more functional due to continuous circulation.

The coronal plane PIV experiments demonstrated novel flow regimes of inhalant flow in *M. galloprovincialis* that have not been previously reported (Fig. 2A). These flow regimes reveal an important concept of non-interacting inhalant and exhalant jet flow behavior. The inhalant flow resembles a low-velocity sink-type 3D flow field that is highly curved at the mussel mantle, while the exhalant flow is more concentrated as a higher velocity jet (Fig. 5). Lower values of energy dissipation are observed at the interface where exhalant and inhalant flows would otherwise interact and result in energy loss hot spots. As such, the highest dissipation rates are localized only at the borders of the exhalant jet core boundary layer. This configuration is beneficial to maintain efficient simultaneous exhalant jet and inhalant flows, reducing high-dissipative interference between these two flow streams. Flow turning angle (Fig. 7) is also important for efficient simultaneous exhalant jet and inhalant flow, and is found to be relatively constant for the sizes studied. We hypothesize that the flow turning angle between inhalant and exhalant flow is critical for energy efficient filtration. Our study suggests that there is a unique angle between exhalant jet and inhalant flow streams, which maintains kinematic similarity.

### Flow control components of mussels

There are numerous functional components in the mussel that are used for inhalant and exhalent flow control. The bulk flow rate, i.e. large amount of water moved by the inhalant and exhalant flows, is modulated through the gape that is adjusted by the shell movement. This can increase and decrease the flow rates of both the exhalant jet and the inhalant flow simultaneously. Inside the shell, we observed that mussels can change the characteristic of exhalant flow from a single core jet to a double core, possibly through conformational changes of the internal exhalant siphon. However, our experimental set-up did not allow us to record the siphon configuration simultaneously with the velocity field, as the siphon is located inside the mussel shells and cannot be seen in the sagittal laser plane (velocity field). However, we have observed the dynamic configuration of the exhalant siphon and illustrated it in a sample movie (Movie 2). The third control element is achieved through the zippering property of the inlet duct of gills that specifically modulates the inhalant flow rate. We observed the coaptation of left and right dorsal edges of mussel gills resembling a fine zipper for suction control (Movie 3). A substantial portion of the flow area can be reduced through this dynamic coaptation region, while the inhalant velocity decreases as 0.8 cm s^−1^, which is related to filtration rate. Another control element is the edge of the mantle, which has plus shaped fringes. They are inside the left and right shells of the mussel as exhalant and inhalant siphons, but there are no fringes at the exhalant siphon. Ciliary structures are also important components of control for exhalant jet flow. The quantity of generated flow can be changed by activation and deactivation of different lateral cilia regions (Seo et al., 2014).

### Exhalant jet flow

While it is observed that the particles larger than 2 μm are retained by the gill filament network of certain mussel species (Vahl, 1972), in our experiments the exhalant jet is fully-seeded with the tracer particles (see Movie 1 of sample raw PIV data). For accurate PIV analyses, the number of particles as low as 10-15 is found to be sufficient for each interrogation window of size, 48×48 pixels. During our experiments only a few of the samples pump partially ‘empty’ water devoid of particles, but these are not included in our manuscript. Further to guarantee reliable PIV analyses for low particle density exhalant jets, we reduced the size of the PIV interrogation window, 96×96 pixels to 48×48 pixels, and demonstrated identical velocity fields.

Peak exhalant flow measurements recorded in the present study for isolated mussels (Fig. 4) are similar to the earlier values presented in the literature (see Frank et al., 2008 and Riisgård et al., 2011), even though mussel species are different. A linear correlation with a low R^2^ value is observed between the peak exhalant velocity and mantle volume; therefore, we organized the mussels in our experiments into three groups based on their volumes. Table 1 shows these three size groups, their corresponding average velocities, and standard deviation values. Mussels with bigger volumes are capable of producing higher exhalant flow velocities (Table 1). In addition, the standard deviation of peak exhalant velocities increases with increasing volume.

Water quality and presence of algal cells may strongly influence the opening degree, and too many cells may cause overloading and result in coughing, production of pseudofaeces, reduction of shell opening and cessation of filtration rate, resulting in wide variation in clearance rate. To prevent measuring non-optimal pumping performance of mussels, we performed auxiliary filtration tests before each experiment for confirmation of the normal steady filtration rate. Long-term monitoring of the peak exhalant jet velocity indicated no periodicity in either the exhalant or inhalant flow, as previously reported (Seo et al., 2014). Time resolved peak exhalant jet velocity also does not show any specific pattern (Fig. 6). Two mussels present different peak exhalant jet behavior. Beyond the periodicity, mussels can change the jet profile from a single core jet to a double core, or visa versa. Changing jet profile was observed in half of the mussels used in these experiments. Jet profile changes should be studied in detail to investigate the exact reason for this phenomenon. While transitionary flow structures are fully captured through the present 2D PIV methodology, a 3D tomographic set-up would elucidate the three dimensional effects. Likewise, the exhalant flow direction is regulated frequently through the exhalant siphon apparatus.

In conclusion, time-lapsed PIV measurements allowed us to understand and quantify the fluid dynamics of exhalant and inhalant flow in *M. galloprovincialis.* Major biological components associated with flow control function were also identified. PIV was applied in two different planes. Coronal plane velocity measurements illustrated that mussels create sink-type inhalant flow, which allowed us to estimate the suction feeding performance of mussels. Pressure gradient is calculated from the measured vector field on the coronal plane, which is used to estimate the suction force. Suction force quantifies the biomechanical characteristic of suction feeding. The velocity field along the sagittal plane was measured to simultaneously investigate the dynamics of exhalant jet and inhalant flow. This measurement plane also allowed us to measure the angle between the exhalant jet and the inhalant flow. FTA is important for optimum pumping as it prevents interaction of inhalant and exhalant flows and found to be independent of mantle size. Simultaneous energy efficient pumping is demonstrated through the hydrodynamic dissipation map computed from the PIV vector field in the sagittal plane (Fig. 8). The peak velocities and dynamics are reported for both flow regimes as a function of size, which provide pumping performance. Mussels should be considered as smart micro-pumps because they can pump their inhalant and exhalant jet flows without any major energy dissipation. Moreover, mussels can change both the velocity and type of exhalant jet, and velocity of inhalant flow using their multiple flow control elements.

## MATERIALS AND METHODS

### Experimental set-up

The Mediterranean mussel, *M. galloprovincialis*, is adopted as a model organism because it is a good representation of the genre and is locally available. Mussels were cultured in a custom aquarium (15×40×30 cm) containing seawater within 15 min after collecting them from the Bosphorus Strait (Istanbul, Turkey) from 6-10 m depth. Mussels were not subjected to any cross-flow in the experimental chamber, which was located on a vibration-isolated table. Exhalant and inhalant flow characteristics of mussels are presented in an inert water environment. We performed experiments in fresh and natural seawater collected from where mussels live. While mussels stay in our experiment chamber in their natural fresh water for a brief period, we verified their viability and performed qualitative filtration rate tests by using organic particles and micro planktons before PIV experiments. PIV experiments were performed when mussels pump water at the maximum filtration rate. We rarely observed coughing and excessive pulsatile jets but these cases were not included in our results and analyses. Experiments were performed on 17 mussels of different sizes. Mussels were released back into the ocean after completing the experiments. The mussels' cavity volumes range from 7 cm^3^ to 34 cm^3^ and the mussels' shell lengths range from 4.88 cm to 7.58 cm.

A cartoon representation of the experimental set-up is sketched in Fig. S1. A shuttered continuous wave laser (LaVision GmbH, Gottingen, Germany) with 1 W output power was used as the laser source at 532 nm wavelengths. Laser guiding arm optics were used to direct the laser to the intended positions and the location of the endpoint of the arm was adjusted through translational stages. A LaVision Imager sCMOS camera (LaVision GmbH, Germany), which is synchronized with the laser, was used to record PIV images using double frame mode. We used Fluoro-Max Dyed red fluorescent polymer microspheres (Thermo Fisher Scientific Inc., MA, USA) with a diameter of 3.2 μm for the PIV experiments performed in the sagittal plane. Fluoro-Max Dyed red fluorescent polymer microspheres with diameter of 1 μ were used for PIV experiments in the coronal plane due to smaller field of view (FOV) and higher magnification. Laser pulse separation between 8000 μs and 14,000 μs was applied, depending on the FOV.

A mussel alignment apparatus was produced in-house from plexiglass with the purpose of aligning the mussel accurately on the laser plane and stabilizing the mussel in its native configuration (Fig. S1B). Mussels were adjusted with an external magnet to precisely align their exhalant and inhalant siphons with a planar (1×1 mm) squared grid that is employed for focusing the camera and PIV calibration.

### PIV protocol and velocity vector field

The raw double-frame particle images were post-processed using Davis 8.2 (LaVision GmbH, Germany) to obtain the velocity profiles similar to our earlier work (Chen et al., 2011, 2013; Pekkan et al., 2016). Five data sets were acquired for each mussel. Each raw data set includes at least 100 image pairs acquired continuously. 100 image pairs are found to be adequate for converged velocity values and also not too long to represent an instantaneous velocity value as a measurement time point. Thus, the presented exhalant jet velocity values in this manuscript are an average of 100 image pairs of corresponding time points of measurement. During the processing of velocity vectors, a mask was applied to segment the mussel cavity. A multi-pass algorithm with decreasing interrogation sizes from 96×96 to 48×48 pixels, both with 50% overlap, was employed. Smoothing and median filters were applied to avoid bad vectors.

### Suction force calculation

Calculation of the pressure gradient provides an estimate of suction force generated by the mussel. Suction force can be calculated from the equation provided in our earlier work (Pekkan et al., 2016). To explore the mussels' suction performance, experiments were conducted in the coronal plane. Suction experiments were performed for three mussels. Three data sets were obtained for each sample and data sets include 100 pairs of images. Time-averaged velocities were considered for pressure gradient calculations through the *y*-momentum equation (Pekkan et al., 2016). The pressure gradient was calculated by assuming 2D flow in the downward suction direction between mussel shells that corresponds to the inside of the mussel in the coronal plane (see Fig. 2A). We employed spatial averaging of pressure gradients in the interspace to obtain smooth trends (Dabiri et al., 2014; Pekkan et al., 2016).

### Energy dissipation rate

The hydrodynamic dissipation rate was computed from the sagittal plane measurements using the following formula:
(3)
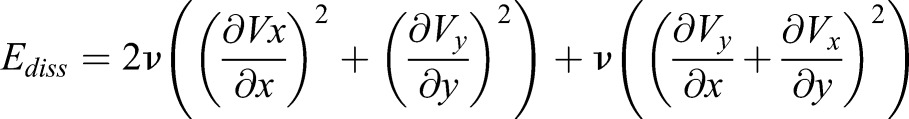
To estimate the dissipation rate in the sagittal plane, we used 2D spatial energy dissipation (Bluestein and Mockros, 1969; Menon et al., 2013).
